# Identification of Distinct Research Gaps that Complement Previous Critiques of Militaristic Language in Relation to Cancer and Other Non-Military Topics

**DOI:** 10.1177/10732748251349935

**Published:** 2025-06-13

**Authors:** Kari Almendingen

**Affiliations:** 1 Department of Nursing and Health Promotion, Faculty of Health Sciences, 158935 Oslo Metropolitan University, Norway

**Keywords:** cancer, terminology, war, media, public discourse, global inequality, due-use technology, mental health, ethics, communication

## Abstract

Militaristic language is pervasive in cancer discourse across media, fundraising, politics, healthcare, and science, despite longstanding critiques from both civilian and military perspectives. Critics argue that framing cancer as a war or battle can lead to feelings of shame and inadequacy, particularly for those with metastatic cancer. This language often diverts focus from prevention and early detection strategies, complicating public perception and understanding of cancer. Two distinct research gaps related to the use of militaristic language in cancer discourse remain unaddressed: the role of dual-use technologies and the perspectives of individuals with wartime experience. Dual-use technologies, initially developed for military applications, have significantly advanced cancer diagnosis and treatment. Yet, their historical and ethical implications are largely absent from public discourse and scientific literature. Awareness of the complex role that dual-use technologies play in cancer diagnostics and treatment, as well as in other societal areas, could influence the prevalence of militaristic language used to describe challenges like cancer, drugs, poverty, and other civil issues. Secondly, studies have not examined opinions on the use of militaristic language among individuals with firsthand wartime experience, — such as civilian victims, military personnel, veterans, pacifists, and aid workers — compared to those without such experience. Both of these omissions may skew findings and overlook diverse perceptions. Addressing these research gaps could foster a more respectful public cancer discourse that takes into account the experiences of affected individuals. This commentary expands on existing critiques, urging professionals to adopt nuanced and inclusive language for cancer and other peaceful topics. Militaristic language is outdated, ethically questionable, and should not be used in science, healthcare, politics, fundraising, or other public contexts.

## Plain Language Summary

Cancer is a major global health issue, causing illness and death worldwide, particularly in low- and middle-income countries. Interestingly, in peaceful regions, people often use warlike language and metaphors when discussing cancer, whether in fundraising efforts, political debates, scientific research, media stories, or other contexts. However, in regions affected by war and conflict, healthcare systems face significant challenges, exacerbating the difficulty of managing cancer effectively. Most scientific studies on this war-themed language in cancer contexts originate from wealthier, peaceful regions. Unlike cancer, diseases like mental illnesses, considered “low-prestige,” are seldom described using war language. This difference highlights how societal values influence the way we talk about, perceive, fund, and prioritize treatment for various illnesses. This paper identifies two major blind spots in the research on war-like language and cancer: first, the lack of input from people who have lived through war, such as civilian victims, war refugees, military personnel including veterans, aid workers, and others; second, the oversight of how dual-use technologies—originally developed for military purposes—play a crucial yet complex role in cancer care. Understanding these technologies might change how we use war language not just for cancer, but for other issues in civilian life too. Using militaristic language to talk about cancer is not only outdated but also raises ethical concerns. It’s time to rethink our words and avoid military language and rhetoric in science, healthcare, politics, fundraising, and other public contexts.

## Earlier Critiques on Using Militaristic Language in Relation to Cancer and Other Non-Military Topics

Militaristic language is pervasive in cancer discourse across media, fundraising, politics, healthcare, and science, despite longstanding critiques from both civilian and military perspectives.^1-11^ Critics argue that framing cancer as a war, with patients positioned as winners or losers in a battle they never chose, can lead to feelings of shame and inadequacy, particularly for those with metastatic cancer who are seen as having “lost the battle.” This language often describes treatments as counterattacks and cancer drugs as weapons, diverting focus from public health strategies like prevention and early detection through screening programs. Such combative narratives may overlook beneficial behaviors that don’t fit the war metaphor, or military rhetoric’, influencing public perception and complicating the understanding of cancer. While some individuals may find empowerment in viewing their illness as a battle, others — especially vulnerable patients —, may adopt this narrative in ways that negatively impact their health behaviour. Additionally, ongoing use of militaristic language can be perceived as oversimplifying and condescending, ignoring the complexities of healthcare in both wartime and peacetime.^1-11^

Since the “war on cancer” slogan was introduced alongside the National Cancer Act of 1971,^3,12^ both cancer survival rates and the general level of education have significantly increased in affluent, peaceful countries. This progress highlights advancements in medical research and public health awareness, contrasting with the initial framing of cancer as an adversary in need of defeat. Despite longstanding critiques of wartime combat language in various civilian contexts, such as the “war on drugs” and “war on poverty,” as well as terms like “killer cells” and the marketing of medicines as “magic bullets,” two critical research gaps related to wartime combat language and increased rearmament remain unaddressed in the literature on militaristic language in cancer discourse and other areas. These gaps underscore the need for further exploration to better understand the impact of such language on perceptions and policies across diverse fields:

## Research Gap nr. 1: The Intricate Legacy of Dual-Use Technologies that Underpins Modern Cancer Diagnosis and Treatment

Modern cancer diagnosis and treatment have been significantly shaped by dual-use technologies that originated from military applications before being adapted for civilian healthcare.^13-18^ The continuous allocation of resources toward rearmament due to geopolitical threats suggests that innovation may remain dual-use, with potential for both positive and negative applications.^19^ However, awareness of the military-civilian links and the ethical responsibilities concerning dual-use technologies remains largely absent from public and scientific discussions on cancer, as well as other peaceful civilian purposes. Nuclear technology, vital for cancer diagnostics and radiation treatments,^20-22^ is for example often discussed without acknowledging its historical association with military projects like the Manhattan Project,^20,23^ as seen in a typical paper exploring the historical background of proton therapy.^21^

Awareness of the complex role dual-use technologies play in cancer diagnostics and treatment, along with their impact on other societal areas, could influence the use of militaristic language to describe civilian challenges such as cancer, drugs, poverty, and other issues in peaceful and affluent regions. This awareness might promote a shift toward more nuanced and precise language that better reflects healthcare realities and technological advancements. However, no studies have yet examined the potential for this awareness to reshape the language used in relation to cancer. Consequently, questions remain about whether individuals informed about the intersections of war, healthcare, and rearmament are more or less inclined to use wartime combat language in relation to cancer and other civilian contexts, and how factors such age, gender and historical knowledge shape their attitudes.

## Research Gap nr. 2: The Perspectives From Individuals With Real War Experiences

Individuals with firsthand wartime experience may interpret militaristic language in cancer discourse differently than those without such experiences.^24^ This interpretation can be influenced by factors such as age, gender, their specific wartime experiences, and access to modern cancer care. However, to date, high-quality studies have not investigated the perspectives of participants with direct wartime experience — such as war refugees, deserters, military personnel, pacifists, veterans, or aid workers — in comparison to those without such experience. Moreover, the views of individuals with varying degrees of conviction about the armed forces, rearmament, or humanitarian aid have also not been thoroughly explored.

A notable example is the scoping review titled “Cancer Experience in Metaphors: Patients, Carers, Professionals, Students,”^11^ which summarized 30 scientific papers, many of them focusing on military language and war metaphors. The research questions of the scoping review were (quote):” (1) What is the extent and nature of published scientific literature on metaphors describing cancer experiences? (2) Within this literature, what metaphors have been identified to portray different aspects of the cancer experience, and how do these metaphors vary among different population groups”.^11^ Conducted by collaborators from several countries, and being the first scoping review on this topic, this review has the potential to be highly cited. However, it overlooks perspectives from those with actual wartime experiences, such as the views of refugees, immigrants, military personnel, veterans, pacifists, aid workers, and humanitarian organizations like the Red Cross. While one study focused on Palestinians in the occupied territories acknowledged that war metaphors might reflect the lived reality in conflict zones,^25^ the scoping review did not delve further into these aspects. Additionally, neither the scoping review nor the included studies addressed the intricate legacy of dual-use technologies that contribute to modern cancer diagnosis and treatment, nor did they explore how this awareness might influence the use of militaristic language in cancer discourse. Furthermore, the scoping review does not consider potential selection bias, skewed findings, or whether militaristic terminology, such as war metaphors, might be perceived as offensive to victims of actual war.

## Research and Future Directions

The present paper is an opinion piece. Nonetheless, an extensive search across various standard medical university databases was conducted, supplemented with internet resources like Google Scholar using a similar iterative search process as described in the scoping review.^11^ The keywords included “dual-use technology,” “war,” “military,” “conflict,” and “armed forces,” which were combined with the keywords mentioned in the review, along with additional keywords not mentioned, such as “war victims,” “veterans,” “aid personnel,” and “military personnel.” While a more extensive search beyond medical databases, including those focused on military research, might reveal relevant scientific literature, the current search did not identify any papers addressing the identified research gaps.

To address the identified research gaps, future studies should investigate whether individuals who understand or are aware of the historical and ongoing connections between war, rearmament, and healthcare are more or less inclined to use militaristic language. These studies could also consider how factors such as age, gender, war experience, and knowledge of war history shape individuals’ attitudes. Furthermore, investigating the perspectives of persons with real war experience regarding the use of war terminology for diseases that can often be prevented and treated, or in other peaceful contexts, is crucial, as these insights are currently lacking. Language and phrases used in military contexts are specifically designed to convey meanings suited to wars and battles. Therefore, future studies could also examine whether military personnel find it inappropriate to use militaristic language outside of military settings.

It has been argued that the historical close relationship between the military and medicine has influenced the language used in medical contexts.^2^ However, this influence is primarily observed in “high-prestige diseases” like cancer,^26^ rather than “low-prestige diseases” such as mental illnesses. Future research could explore how societal and cultural values shape the language used for different illnesses and analyze the use of militaristic language in high- vs low-prestige diseases.

According to the United Nations, a quarter of humanity currently lives in regions affected by conflicts and war. Future language studies could focus on developing an inclusive global language for cancer, potentially by explicitly naming different types and stages of the disease. As previously suggested: “Why not call treatments by their proper names or use less belligerent comparisons at least?”.^2^

In addition to addressing research gaps, integrating inclusive health communication into student curricula could empower students to become change agents, advocating for non-violent language in discussions about cancer and other topics. This approach could also help shift norms away from militaristic terminology toward a more constructive way of communicating, extending beyond medicine and health care.

## Conclusion

In conclusion, the lived realities of conflict-affected populations and the awareness of the complex military legacy in cancer advancements are often overlooked in the central literature addressing militaristic language in relation to cancer. These research gaps can lead to incomplete or biased conclusions in scientific studies. By identifying these gaps, this commentary contributes to and expands upon existing critiques, reinforcing the argument that professionals — including policymakers, community leaders, cancer charities, fundraising organizations, health professionals, and others — should abandon the use of militaristic language in cancer discourse and beyond. This commentary not only highlights the missing elements in current literature but also builds on previous critiques, advocating for a more nuanced and respectful language in relation to both cancer and other peaceful topics.
